# Telomerase subunit Est2 marks internal sites that are prone to accumulate DNA damage

**DOI:** 10.1186/s12915-021-01167-1

**Published:** 2021-11-20

**Authors:** Satyaprakash Pandey, Mona Hajikazemi, Theresa Zacheja, Stephanie Schalbetter, Matthew J. Neale, Jonathan Baxter, Victor Guryev, Andreas Hofmann, Dieter W. Heermann, Stefan A. Juranek, Katrin Paeschke

**Affiliations:** 1 University of Groningen, University Medical Center Groningen, European Research Institute for the Biology of Ageing, 9713 AV Groningen, Netherlands; 2 Clinic of Internal Medicine III, Oncology, Hematology, Rheumatology and Clinical Immunology, University Hospital Bonn, Bonn, Germany; 3 Department of Life Science, University of Sussex, Brighton, UK; 4 Institute for Theoretical Physics, University of Heidelberg, Philosophenweg 12, 69120 Heidelberg, Germany

**Keywords:** DNA damage, Genome stability, Telomerase, Yeast

## Abstract

**Background:**

The main function of telomerase is at the telomeres but under adverse conditions telomerase can bind to internal regions causing deleterious effects as observed in cancer cells.

**Results:**

By mapping the global occupancy of the catalytic subunit of telomerase (Est2) in the budding yeast *Saccharomyces cerevisiae*, we reveal that it binds to multiple guanine-rich genomic loci, which we termed “non-telomeric binding sites” (NTBS). We characterize Est2 binding to NTBS. Contrary to telomeres, Est2 binds to NTBS in G1 and G2 phase independently of Est1 and Est3. The absence of Est1 and Est3 renders telomerase inactive at NTBS. However, upon global DNA damage, Est1 and Est3 join Est2 at NTBS and telomere addition can be observed indicating that Est2 occupancy marks NTBS regions as particular risks for genome stability.

**Conclusions:**

Our results provide a novel model of telomerase regulation in the cell cycle using internal regions as “parking spots” of Est2 but marking them as hotspots for telomere addition.

**Supplementary Information:**

The online version contains supplementary material available at 10.1186/s12915-021-01167-1.

## Background

Telomeres are multi-protein complexes at the ends of eukaryotic chromosomes. A major function of telomeres is to protect the integrity of the genome. The length of the telomeres is critical for survival as shortening of telomeres leads to senescence and eventually cell death [1]. Telomerase, a highly specialized reverse transcriptase, is responsible for maintaining telomere homeostasis using an intrinsic RNA subunit as a template [2]. Telomerase upregulation is a characteristic signature for cancer cells and genome instability [3]. Telomere structure, function, and maintenance *via* telomerase are conserved throughout eukaryotes [4]. In *Saccharomyces cerevisiae*, telomerase is composed of three proteins, Est1, Est2, Est3, and an RNA subunit, TLC1. The catalytic subunit of telomerase, Est2, is expressed throughout the cell cycle and associates with telomeric regions primarily during late S-phase [5]. Two different pathways recruit telomerase to the telomeres in G1 and S/G2 phase. In G1 phase yKu heterodimer (Ku70, Ku80) interacts with Sir4 and binds to TLC1. This is a prerequisite for the Est2-TLC1 interaction and accumulations of telomerase at telomeres. However, telomerase is devoid of Est1 and Est3 in G1 phase and remains inactive. In S/G2 phase, Cdc13 recruits Est1, which in turns allows the recruitment of Est3. Est1 is required for full activation of telomerase [6–10]. Multiple unbiased approaches have yielded a list of proteins involved in regulating telomerase function, but the mechanisms that recruit and activate telomerase are still not completely known [5, 11–13].

Genome stability is constantly challenged and efficient repair mechanisms are essential to maintain genome integrity [14–16]. Defects in the repair pathways result in increased genome instability caused by deletions, mutations, end-to-end fusions, translocation, and de novo telomere addition at internal sites [14]. De novo telomere addition by telomerase at DNA double-strand breaks (DSB) is hazardous for the cell, because all genetic information distal to the DSB is lost [16–19]. Studies in yeast and human suggest that telomerase components are not associated with the telomere throughout the cell cycle and the catalytic subunit of telomerase itself or associated proteins perform a function at internal regions [8, 20–27]. For example, single-molecule image tracking of human telomerase revealed a three-dimensional diffusion model wherein telomerase makes multiple transient and stable contacts with telomeres during different cell cycle phases [8, 27]. Multiple interactions can be observed throughout S phase before telomerase binds to the 3′ overhang of the chromosome ends [27]. Microscopic imaging in yeast demonstrated that TLC1 segregates to different cellular locations during different cell cycle stages to prevent de novo telomere addition [24]. Single molecule imaging showed that TLC1 remains in the nucleoplasm in G1/S phase and the nucleolus in G2/M phase. This segregation is lost under DNA damage conditions in *rad52Δ* cells in which TLC1 localizes at DSBs and leads to de novo telomere addition. Multiple proteins such as Pif1, Cdc13, and the SUMO ligase Siz1 are involved in regulating telomerase action at DSBs [24–26, 28–31]. Additionally, genomic sequencing of bleomycin-treated yeast cells revealed additional regions where telomere addition occurs in the genome [24]. Specific subsets of genomic sequences termed as sites of repair-associated telomere addition (SiRTAs) have been identified where de novo telomere addition occurs upon a DSB [25]. Genetic assays using an HO endonuclease system demonstrated that de novo telomere addition at these sites depends on Cdc13 and Rap1 [25]. Although these sites contain a bipartite structure, a global prediction and validation of SiRTAs under different genetic and biochemical conditions is still missing.

Considering these findings, it is essential to reveal whether, when, and where telomerase localizes to specific internal sites and what is the impact of this interaction on genome stability. Here, we provide a comprehensive map of the global occupancy of Est2 within the genome for the first time. Interestingly, Est2 binds to multiple internal genomic loci, termed non-telomere binding sites (NTBS). Using differential cell cycle analysis, we revealed that Est2 binds to NTBS independent of Est1 and Est3 in G1 and G2 phases. In the past, different models have been proposed to explain how telomerase is recruited to the telomeres [25, 27, 29, 32–38]. Using Hi-C analysis, we found that NTBS are in closer proximity to telomeres than expected by random chance, suggesting a potential correlation between chromatin organization and telomerase sequestration in different cell cycle phases. Because Est2 binds independently of other known telomeric factors to NTBS, telomerase is inactive at these sites. However, NTBS regions are prone to DSBs and upon global DNA damage Est2 recruits Est1 and Est3 and active telomerase assembles, resulting in telomere addition at NTBS. We propose a model in which Est2 binds to multiple guanine-rich sites across the genome where it is parked in an inactive form. This renders NTBS a hotspots for telomere addition and genome instability.

## Results

### Est2 binds to non-telomeric regions within the genome

In order to determine regions of telomerase action within the genome, we monitored the genomic occupancy of Est2 in *S. cerevisiae* using a strain wherein Est2 was internally tagged at its C-terminus with 13 x Myc (Est2-Myc13). Yeast cultures expressing Est2-Myc13 were crosslinked with formaldehyde and subjected to chromatin immunoprecipitation (ChIP). DNA bound to Est2-Myc13 and input DNA were fluorescently labeled and hybridized to a whole-genome DNA microarray (ChIP-chip) (Agilent). The binding sites were identified from the median standardized array values (across biological triplicates) using the ChIPOTle 2.0 program with a significance cut-off of 0.05. The experiment was repeated 5 times and only regions that could be identified in at least three biological replicates were annotated as *bona fide* Est2 targets.

**Fig. 1:**
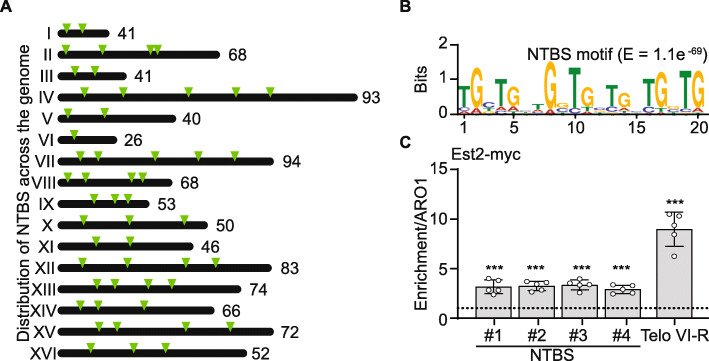
Global occupancy of Est2 across the yeast genome. **A** The distribution of Est2 occupancy across the *S. cerevisiae* genome. Each triangle represents a non-telomeric binding site (NTBS) of Est2 on a chromosome. All the sites were present in at least 3 out of 5 independent experiments. Note, less triangles are visible on the cartoon because of the resolution of the graphic. Multiple regions that are located at close to each other or as clusters are depicted as one arrow. **B** MEME motif of NTBS regions. The binding sites displayed an enriched TG-richness similar to yeast telomeric regions. (*E*-value 1.1e−069) **C** ChIP-qPCR of four different NTBS regions (see Additional file 1: Table S1 for specification of the region). As a positive control, Est2-binding to telomere VI-R was plotted (Telo-VI-R). Reported ChIP values are normalized to input and ARO1 (non-telomeric control). Data are represented as mean ± standard error mean (SEM) of *n* = 5 biological replicates unless stated otherwise. Statistical significance was compared to ARO1 levels and determined using Student’s *t*-test. **p-value < 0.01 and ***p-value < 0.001

After subtraction of telomeric sequences, Est2 ChIP-chip analysis led to the identification of 978 NTBS (see Additional file 1: Table S1 for a list of NTBS) (Fig. 1A (graphical illustration of regions harboring NTBS) and Additional file 2: Fig. S1A that illustrates Est2 binding peaks of four different regions: NTBS#1-NTBS#4). Bioinformatics analysis revealed that these sequences are significantly more G-rich than the average GC content of the yeast genome (NTBS: 52% GC; yeast genome: 38% GC; *p*-value < 0.001). MEME motif analysis displayed a characteristic TG-richness, that despite presenting other nucleotides, corresponds the motif of telomeric repeats in yeast (Fig. 1B, E-value = 1.1e^−69^). We computationally correlated NTBS peaks to annotated genomic regions (annotated by *S. cerevisiae* genome database (SGD) such as autonomously replicating sequence (ARS), promoter) or binding sites of specific proteins. Our analyses showed that NTBS overlap significantly with regions that are also bound by known telomerase regulatory factors: G-quadruplex (G4) regions [39] (35/978 *p* < 0.0001), R-loops [10, 40] (84/978 p < 0.0001), and Pif1-binding sites [41] (361/978 *p* < 0.0001). Also these regions are linked to genome instability as indicated that these sites overlap with sites high in DNA polymerase II (DNA Pol II) occupancy—marking regions where DNA replication stalls in wild type [41] (354/978 p < 0.0001) and in *pif1-m2* cells (430/978 p < 0.0001) [41] as well as sites that are highly linked to DNA damage as indicated by a strong γ-H2A signal [39] (294/978 *p* < 0.0001) (Additional file 2: Fig. S1B-G). In *pif1-m2* no nuclear Pif1 is present, only mitochondrial Pif1 is expressed. Furthermore, correlation analysis revealed that > 85% of NTBS significantly overlap with open reading frames (ORFs, *p*-value < 0.001, of which 56 genes are involved in telomere maintenance and homeostasis (Additional file 3: Table S2). Note, it is not clear to this point if Est2 binding to these ORF is relevant for telomere function or biology.

Next, we validated Est2-binding to intrinsic sites by ChIP followed by quantitative PCR (ChIP-qPCR) using primers directed against 4 different NTBS (NTBS#1-NTBS#4). Here, and in all subsequent ChIP experiments, we used the right telomere on chromosome VI (Telo VI-R) as a positive control and *ARO1*, a known region low in telomere-binding proteins, as a negative control [42]. ChIP-qPCR analysis of Est2 revealed a robust and significant binding to all tested NTBS (Fig. 1C). Est2-binding was 2–3-fold enriched in comparison to the negative control *ARO1*.

### Est2-binding to NTBS is regulated throughout the cell cycle

**Fig. 2:**
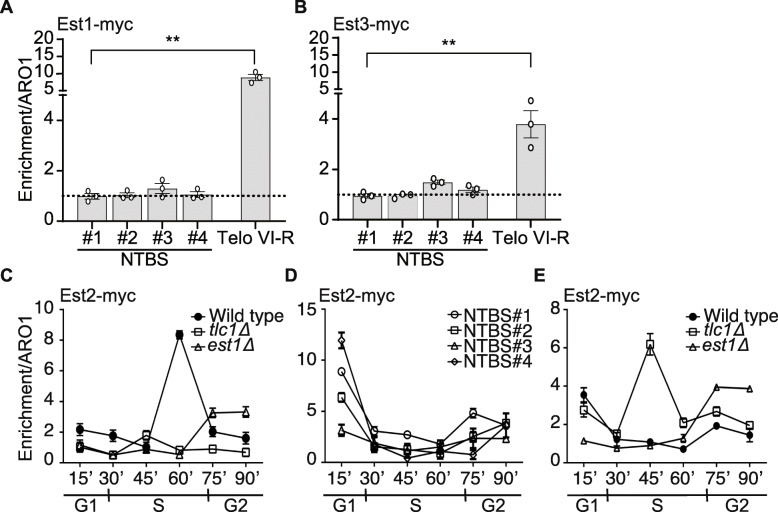
Est2-binding to NTBS does not depend on Est1 and TLC1. ChIP analysis of Est1 and Est3 to four NTBS and one telomere (VI-R) (**A**, **B**). **A** Est1-binding to NTBS, ARO1, and Telo-VI-R regions. Bars represent enrichment over ARO1. Data are represented as mean ± SEM for *n* = 3 biological replicates. Statistical significance compared to untreated cells were determined using Student’s *t*-test. *p-value < 0.05 and **p-value < 0.01. **B** Est3-NTBS binding. **C** Est2-binding was monitored in synchronized cultures. For this, cells were synchronized using α-factor and released in the cell cycle. Binding was monitored every 15 min of release into the cell cycle. FACS analysis was performed to analyze the cell cycle stage of synchronized cells depicted in Additional file 4: Fig. S2A. The graph represents Est2-binding to telomere VI-R in wild type background (closed circles, in absence of TLC1 (open squares) and in the absence of Est1 (open triangles). **D** Est2-binding to NTBS #1-#4 in wild type background. **E** Representative data of Est2-binding to NTBS#1 in wild type, *tlc1Δ*, *est1Δ* (Additional file 4: Fig. S2B-D for NTBS #2 -#4). The data plotted are standard mean ± standard error for *n* = 3 replicates

At telomeres Est2 functions in a complex with Est1 and Est3 [5, 43]. In vivo data shows that all components need to be present for an active telomerase holoenzyme [6, 43–46]. To determine whether telomerase is active at NTBS, we asked if only Est2 or the whole telomerase holoenzyme is binding to NTBS. We analyzed the binding of Est1 and Est3 to four different NTBS in asynchronous yeast cells by ChIP-qPCR (Fig. 2A, B). Both, Est1 and Est3, were tagged internally with 13xMyc. After crosslinking, protein binding was monitored by ChIP-qPCR. These analyses revealed that neither Est1 nor Est3 bind significantly to these NTBS, indicating that Est2 binds alone and thus is likely not active at NTBS.

Est2-binding to telomeres changes in a cell cycle-specific manner [5, 7, 42]. We asked whether Est2-binding to NTBS is also cell cycle-dependent. We synchronized yeast cells in G1 with α-factor and released them into S-phase as performed previously [7]. Cell cycle progression was monitored by fluorescence-activated cell sorting (FACS) (Additional file 4: Fig. S2A). Est2-binding in different cell cycle phases was monitored by ChIP-qPCR. Est2-binding peaks at the end of S phase at telomeres, which agrees with published data [7] (Fig. 2C, black circles). On the contrary, Est2-binding to all four NTBS peaked in G1 and late S/G2 phase (Fig. 2D). Note, the Est2-binding to NTBS is less strong as to telomeres.

At telomeres, Est2-binding depends on the presence of Est1, Est3, and TLC1 [6, 7, 9, 42, 45, 46]. To test, if Est2-binding to NTBS changes in the absence of telomerase subunits (Est1, TLC1), we performed cell cycle-dependent ChIP-qPCR in *est1Δ* and *tlc1Δ* backgrounds. At telomeres, Est2-binding is reduced when either TLC1 or Est1 is absent (Fig. 2C, white squares and triangles). This agrees with previously published data [42]. However, at NTBS Est2-binding is enhanced (9.6-fold) in late S/G2 phase in *est1Δ* cells (Fig. 2E, Additional file 4: Fig. S2B-D, white triangles). In *tlc1Δ* cells Est2-binding to NTBS was significantly elevated across all cell cycle stages with a strong peak in mid-S phase (Fig. 2E, Additional file 4: Fig. S2B-D, white squares). We speculate that without TLC1 Est2 no longer binds to telomeres and consequently more Est2 is “free”, which results in more Est2-binding to NTBS.

### Est2 binds to NTBS independently of known telomere-binding proteins

**Fig. 3:**
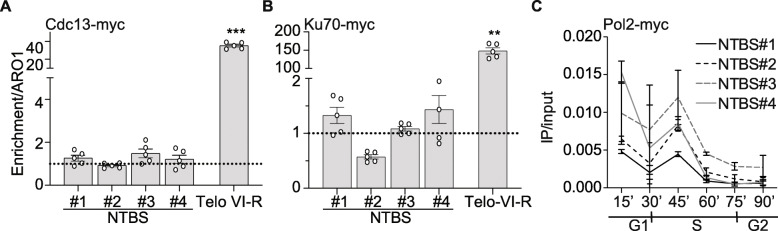
Est2 is recruited via an alternative pathway to NTBS. **A** Cdc13 was tagged internally and binding to four NTBS and telomere VI-R was monitored by ChIP-qPCR in asynchronous cultures (Additional file 5: Fig. S3A-B for synchrony ChIP-qPCR of Cdc13). **B** Ku70 is tagged internally and binding to NTBS and telomere VI-R was monitored by ChIP-qPCR in asynchronous cultures. ChIP values are normalized to input and ARO1 (non-telomeric control). Data are represented as mean ± SEM of *n* = 5 biological replicates unless stated otherwise. Statistical significance was compared to ARO1 levels and determined using Student’s *t*-test. **p-value < 0.01 and ***p-value < 0.001. **C** DNA Pol2 occupancy was monitored throughout the cell cycle to NTBS and telomeres. Representative data of DNA Pol2-binding to NTBS#1-4, normalized to input

Cdc13, Est1, and the heterodimer yKu70/80 regulate telomerase recruitment to telomeres. They are essential for telomere maintenance [7, 35, 42, 47, 48]. Cdc13 and Est1 recruit Est2 during S/G2 phase, while yKu70/80 is required for Est2-binding at telomeres during G1 and early S phase and significantly contributes to the association of Est2-binding in S/G2 phase at telomeres [7, 35, 42, 47, 48]. Therefore, we aimed to understand if either Cdc13 or yKu heterodimer support Est2-binding to NTBS. We first analyzed Cdc13- and yKu70-binding to NTBS. Both proteins were tagged internally and their binding to NTBS was measured by ChIP-qPCR (Fig. 3A, B). We observed little to no binding of yKu70 (0.7-1.7-fold binding/ARO1) or Cdc13 (1-3-fold binding/ARO1) to NTBS (Fig. 3A, B). Note, at telomeres, Cdc13 is nearly 30-fold and yKu70 over 100-fold enriched over ARO1 (Fig. 3A, B). Thus, it can be concluded that both proteins do not play a major role in mediating Est2-binding at NTBS. Although Cdc13 binds throughout the cell cycle its binding peaks during S/G2 phase [42]. To rule out that ChIP in asynchronous cells yields false interpretations, we also performed the Cdc13 ChIP experiments in synchronized cells. Nevertheless, similar results were obtained that showed only minor binding of Cdc13 to NTBS (Additional file 5: Fig. S3A-B).

### Recruitment of Est2 to NTBS

In addition to Cdc13 and Ku70, other proteins and mechanisms have been postulated to regulate the recruitment of Est2 to telomeres. Among them are Pif1 [28, 29, 31], Mlh1 [49, 50], R-loop formation, and Telomeric repeat-containing RNA (TERRA) [51–53], RNase P components [13], and Rad51-Rad52 [23]. To reveal if one of these potentially regulatory factors contributes to Est2-binding, we monitored Est2-binding by ChIP-seq in the absence of these factors. In summary, no significant changes in Est2-binding were observed in *pif1-m2*, after the deletion of Mlh1 (*mlh1Δ*) or the reduction of R-loops by the overexpression of RNase H1 (Additional file 6: Fig. S4A-C). The yKu70/80 heterodimer binds to telomerase in G1 phase in a Sir4-dependent manner [37, 54, 55]. Sir4 is important for the telomere position effect (TPE), which may also contribute to Est2-binding to NTBS. However, Est2-binding to NTBS was not altered in the absence of Sir4 (Additional file 6: Fig. S4D).

In addition to these factors, it has been shown that the heterochromatic state of telomeres alters the access of telomerase to the telomeres. Sin3 is a component of the histone deacetylase complex that is responsible for the deacetylation of the core histones and effects heterochromatinization [56]. To test if the heterochromatic state of NTBS changes Est2-binding, we analyzed Est2-binding in *sin3Δ* cells by ChIP-qPCR. However, changes in *sin3Δ* had only minor and no-significant effect on Est2-binding to NTBS (Additional file 6: Fig. S4E).

We demonstrated that the recruitment of Est2 did not correlate to known recruitment factors of the telomere. Next, we investigated other published models such as the “replication fork” model. In this model, telomerase co-migrates with the replication fork [20, 36]. NTBS overlap with regions that are marked as replication fork pausing sites and we tested if replication fork pausing correlates with Est2-binding. If replication pauses cause Est2-binding, we assumed that the timing of Est2-binding to NTBS should mimic replication fork progression. We tagged the catalytic subunit of the leading strand polymerase (DNA Pol2) and used its occupancy as a measurement of replication fork pausing [41, 57]. We synchronized yeast cells and measured the binding by ChIP-qPCR. The results indicated that the timing of DNA Pol2-binding and Est2-binding does not correlate with each other (Fig. 3C). Our data indicate that replication fork pausing is not the cause for Est2-binding to NTBS.

**Fig. 4:**
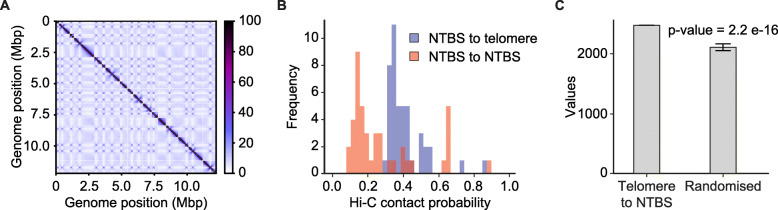
Hi-C data. **A** Representative Hi-C contact map of interchromosomal contacts plotted at 5-kb resolution. **B** Histogram of the Hi-C contact probability of NTBS-NTBS and NTBS-telomeres interaction. Hi-C data show that NTBS sites are closer to each other than to telomeres in roughly 86 out of 100 cases. **C** Telomere-NTBS interactions are statistically significant than random chance (*p*-value = 2.2e−16)

Telomerase-binding to telomeres follows a three-dimensional model wherein telomerase makes multiple contacts with the chromosomes before binding to the telomeric regions [27]. To assess whether the three-dimensional organization of chromosomes has a role in the binding of Est2 to NTBS we performed chromosome conformation capture using the Hi-C technique. Wild type cells were subjected to Hi-C as described [58] and the resulting libraries were sequenced to determine the interactions between NTBS-NTBS and NTBS-telomeric regions (Fig. 4A). We analyzed whether for a given NTBS the other binding sites are on average (mean Hi-C contact probability) closer to another NTBS or to telomeric regions (max Hi-C contact probability). Our bioinformatics analyses revealed the mean Hi-C contact probability of NTBS-NTBS interactions is 841/978 (86%) (Fig. 4B). 137/978 (14%) NTBS regions are closer to telomeric regions. Importantly, an iteration analysis showed that the NTBS are significantly closer to telomeres than randomized control regions (*p*-value 2.2e^−16^) (Fig. 4C). These data suggest that the chromatin organization dictates Est2-binding to NTBS.

### DNA damage repair is not supporting Est2-binding to NTBS

**Fig. 5:**
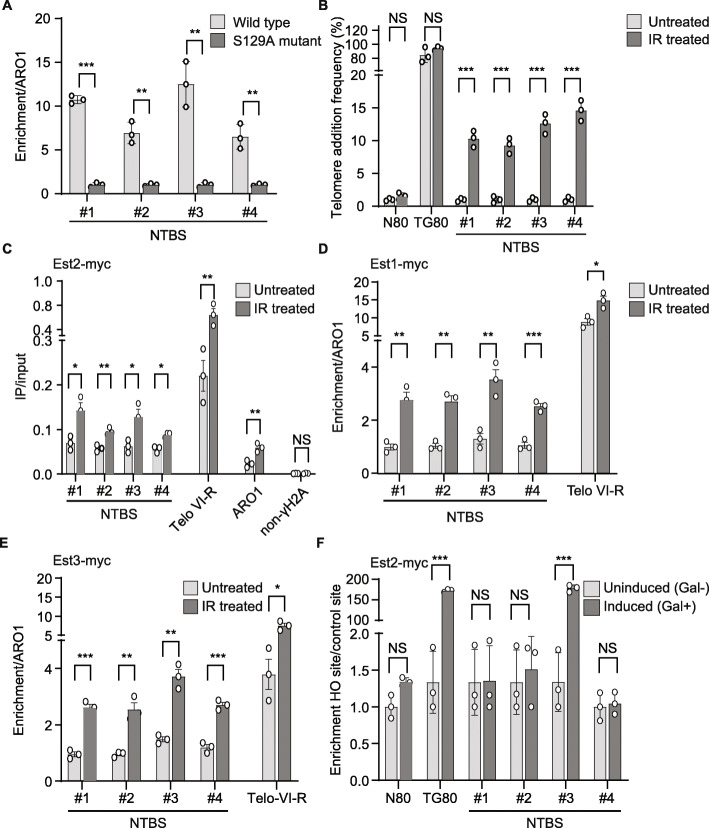
Est2 binding is affected by DNA damage. **A** ChIP-qPCR of γ-H2A-binding to NTBS regions demonstrating their DNA damage prone nature. H2A-binding to four NTBS (#1-#4) and compared to S129A mutant (no γ-H2A phosphorylation). Data plotted are mean ± SEM for *n* = 3 biological replicates with wild type (light grey bars) and S129 mutant (dark grey bars) conditions. Statistical significance compared to S129 mutant conditions were determined using Student’s *t*-test. **p-value < 0.01. **B** Telomere addition frequency was determined in undamaged (light grey bars) and in damage (IR, dark grey bars) and was calculated as described before [26]. For IR treatment, cells were irradiated at 20 Gy before crosslinking and immunoprecipitated using the standard procedures mentioned in the methods. Telomere addition frequency was measured using a genetic assay based on loss of distal *LYS2* gene (resistance to α-aminoadipate). TG80 and N80 were used as positive and negative control. TG80 contains 80 bp TG_1–3_ ; N80 contains 80 bp lambda DNA. **C–E** ChIP analysis of Est2, Est1, and Est3 to four NTBS and one telomere (VI-R) in undamaged (light grey bars) and damaging (IR, dark grey bars) conditions). For IR treatment, cells were irradiated at 20 Gy before crosslinking and immunoprecipitated using the standard procedures mentioned in the methods. **C** Est2-binding to NTBS, *ARO1* and non-γ-H2A regions. Data plotted are IP/Input values represented as mean ± SEM of *n* = 3 biological replicates. Statistical significance compared to untreated cells were determined using Student’s *t*-test. *p-value < 0.05 and **p-value < 0.01. **D** Est1-NTBS-binding. ChIP is normalized to ARO1 and represented as mean ± SEM. **E** Est3-NTBS-binding in undamaged (light grey bars) and damaging (IR, dark grey bars) conditions. Bars represent enrichment over ARO1. Data are represented as mean ± SEM for *n* = 3 biological replicates. Statistical significance compared to untreated cells were determined using Student’s *t*-test. *p-value < 0.05 and **p-value < 0.01. **F** Quantification of Est2-binding upon induction of cleavage at the HO site. Est2-binding by ChIP to NTBS near HO cut sites was monitored before (light grey bars) and after induction (dark grey bars) of HO endonuclease. Data were plotted as mean ± SEM of *n* = 3 biological replicates. Statistical tests were performed by comparing induced to uninduced conditions and were determined using Student’s *t*-test. ** *p*-value < 0.01

Telomerase can act at DSBs under specific conditions [25, 26, 29, 30, 59–61]. In many cancers, telomerase is reactivated at telomeres as well as at internal sites and these events cause genome instability and can drive tumorigenesis [62–67]. In addition, telomeres are known to be hotspots to accumulate DNA damage, as indicated by high levels of γ-H2A. γ-H2A is a histone modification (phosphorylation) that occurs in response to DNA breaks [68]. We speculated that NTBS, which show similarities to telomeric G-rich repeats, are also DNA damage prone. We performed bioinformatic analyses that revealed a significant overlap between a DNA damage marker (phosphorylated histone H2A, γ-H2A) with NTBS sites (Additional file 2: Fig. S1G). NTBS, like telomeres, are significantly enriched in regions that accumulate high levels of γ-H2A (294/978) [*p*<0.0001] (Additional file 2: Fig. S1G). ChIP-qPCR using an anti-γ-H2A antibody was performed to confirm these results. We observed a 5–12-fold higher binding of γ-H2A to NTBS in comparison to a H2A S129A mutant, which cannot be phosphorylated (Fig. 5A) [69].

Due to high levels of γ-H2A, we conclude that NTBS regions are vulnerable to accumulate DNA damage. In yeast, DNA damage is mainly repaired by homologous recombination (HR). However also telomerase can act at DSB, which should be avoided to preserve genome stability. Rad52 is a critical protein for HR in yeast [23, 70]. We examined whether the enrichment of Est2 is altered in the absence of Rad52. At telomeres, Est2-binding was 2-fold reduced in *rad52Δ*. However, at NTBS, we did not detect significant changes in Est2-binding in *rad52Δ*, suggesting that Est2-binding to NTBS is not HR dependent (Additional file 7: Fig. S5A).

To further address whether Est2-binding to NTBS causes telomere addition, we quantified telomere addition using a telomere healing assay [26, 60] (Fig. 5B, Additional file 7: Fig. S5B). We speculated that telomerase is not active at this site, because Est1 and Est3 are not present (Fig. 2). A lack of de novo telomere addition would further support that telomerase is not active at NTBS. Telomere addition was observed if telomeric repeats (TG80) were added next to an HO endonuclease cut site. If a random sequence (called N80) was near the HO cut site no telomere addition occurred (Fig. 5B, D, Additional file 7: Fig. S5B). To address if NTBS act like telomeric sequences and enhance telomere addition, we cloned four different NTBS at the same position adjacent to an HO cut site (see Additional file 8: Table S3 for list of the NTBS). Addition of galactose led to the induction of the HO endonuclease and subsequent processing at the HO cut site. In dependency to the repair at the HO cut site, the cell either loses or retains the adjacent marker (*LYS2*). If the break is repaired by telomere addition, the *LYS2* marker is lost. If the break is repaired via non-homologous end joining, the *LYS2* marker is retained (Fig. 5B, Additional file 7: Fig. S5B). After the break induction colony formation was monitored. Colony counting revealed that no telomere addition was monitored at 4/4 NTBS regions whereas 100% telomere addition was observed at TG80 controls (Fig. 5B). These data confirm binding of inactive Est2 to NTBS.

### NTBS are hotspot for genome instability

Cancer is connected to increased telomerase activity and genome instability [71]. In multiple cancers, telomerase is activated and telomere addition can be observed at many internal sites, which drives genome instability, aneuploidy, and polyploidy [72–76]. To test if increased genome instability leads to telomerase activation at these sites, we treated cells with ionizing gamma radiation (IR) to increase overall DNA damage in cells. Following treatment, we monitored Est2-binding by ChIP-qPCR to NTBS and controls. We selected 20 Gy, which causes global DNA damage but leaves 80–90% of the cells viable [77]. Upon IR treatment Est2-binding to NTBS was significantly enhanced (1.5–3-fold) compared to untreated control cells (Fig. 5C). However, Est2-binding to *ARO1* also increased 2-fold but binding remained the same to a region devoid of γ-H2A-binding previously identified by genome-wide approaches [39] (Fig. 5C). We concluded that although NTBS have high levels of γ-H2A, enhanced global DNA damage stimulates Est2-binding to NTBS and leads to Est2-binding to additional internal sites (for example, *ARO1*).

NTBS are prone for DNA damage (Fig. 5A, B, Additional file 7: Fig. S5A-B) and Est2-binding is stimulated upon IR (Fig. 5C). To test if an active telomerase complex assembles during DNA damage at NTBS, we performed ChIP analyses with Est1 and Est3 after IR treatment. Interestingly, both proteins bind to NTBS upon IR treatment 2–4-fold more compared to untreated control cells, supporting the conclusion that in untreated cells telomerase enzyme is inactive, but upon damage the holoenzyme assembles (Fig. 5D, E, gray bars). To check if elevated binding of Est1 and Est3 are mediated via enriched binding of Cdc13 or Ku70 after IR treatment, we performed ChIP-qPCR after IR treatment. Asynchronous cells were treated with IR and binding of Cdc13 and Ku70 was monitored by ChIP-qPCR. Analysis revealed that upon IR treatment, no significant binding was observed for neither Cdc13 and Ku70 to NTBS (Additional file 5: Fig. S3C-D). These data indicate that upon damage, Est1 and Est3 are recruited to NTBS because of the presence of Est2. Because we can exclude that Est1 and Est3 are recruited by similar mechanisms as to telomeres, it is not clear how they are recruited to NTBS-Est2.

We next wanted to determine if a specific break at the NTBS stimulates Est2-binding similar to IR treatment. We performed ChIP-qPCR after HO induction to quantify Est2-binding to NTBS [60]. HO induction resulted in a specific cleavage near the NTBS as opposed to IR treatment wherein global DNA damage occurs. ChIP-qPCR quantification revealed that Est2 associates to NTBS near the HO sites but binding of Est2 is not stimulated upon HO induction apart from one NTBS site (Fig. 5F). This indicated that a threshold of global damage is required for Est1- and Est3-binding to NTBS regions (Fig. 5D, E). Next, we investigated if increased global DNA damage not only results in more Est1-, Est2-, and Est3-binding, but also leads to telomerase activation. To monitor telomere addition, we used the previously described telomere addition assay where we inserted NTBS near HO sites after IR treatment (see Additional file 7: Fig. S5B). Colony formation showed that upon increased global DNA damage telomere addition can be monitored at 4/4 NTBS sites (Fig. 5B). We could demonstrate that NTBS are parking spots for Est2 in normal conditions, but hotspots for telomere addition if overall DNA damage increases in these cells. We predict that these sites are marked for telomere addition due to the presence of Est2.

## Discussion

We identified internal DNA binding sites of Est2 and addressed the questions: how Est2 is recruited and localized to NTBS. Multiple studies have focused in the past on telomerase recruitment [5], and its activity and regulation at telomeres vs. DSB [16, 29, 78]. The here determined internal binding regions of Est2 binding leads to the hypothesis that internal Est2 binding sites are prone for telomere addition and cause genome instability.

Our data demonstrates that Est2 binds to over 900 NTBS. These sites are TG-rich and which has similarities to telomeric repeats in *S. cerevisiae* (Fig. 1). The cell cycle specific binding pattern of Est2 either to telomeres (S phase) or to NTBS (G1/G2 phase) suggests a cell cycle specific recruitment process. Therefore, we investigated if Est2 is recruited to NTBS via similar mechanisms as to telomeres. We observed that Est2 is not recruited to NTBS via similar mechanisms than it is to telomeres (Cdc13, Ku70/80, R-loops, Pif1, Mlh1) (Fig. 3, Additional files 5, 6: Fig. S3, S4). Furthermore, neither HR (Additional file 7: Fig. S5B), heterochromatin formation, or replication pausing [20, 36] (Additional file 6: Fig. S4) is the cause of Est2-binding to NTBS. Est2-binding to NTBS is also TLC1-independent (Fig. 2E). But we observed enhanced binding of Est2 to NTBS when TLC1 is missing in the cells (Fig. 2E). We anticipate that without TLC1, Est2 is no longer efficiently recruited and anchored to telomeres and therefore “free” to bind to other (internal) G-rich regions. Our data suggest that the three dimension organization of the chromatin dictates and supports Est2 localization to NTBS, which we indeed could show in Hi-C analysis (Fig. 4). Telomere looping maintains the telomere position effect (TPE), leading to the repression of transcription of telomere-adjacent genes [34]. How telomere looping is mediated, if its function is only to maintain the TPE, and whether Est2 is involved in this process are not clear, yet. It is likely that other DNA structures support this looping and sequestering of Est2 to NTBS. We speculate that G4-G4 interaction might support this looping, because telomeres as well as NTBS are regions prone for G4 formation. NTBS overlap to published G4 regions (*p*<0.0001) [39] (Additional file 2: Fig. S1B). In addition, G4 formation has also been discussed to promote long-range DNA interactions [79–81], which makes it tempting to speculate that G4 might support Est2-binding to NTBS. The function and relevance of G4 structures for telomere maintenance is a long ongoing discussion. Multiple data show how G4 formation can alter different aspects of telomere maintenance [82, 83], such as binding of telomere binding proteins [84], altering telomerase function [83–87], or the telomere organization within the nucleus [84, 85, 88].

Telomere addition at DSB contributes to genome instability and should be always prevented. Our finding that Est2 binds under normal wildtype conditions to internal sites is counterintuitive and raises the question of telomere addition at NTBS and their impact on genome stability. In unchallenged yeast cells Est2 binds to NTBS without the telomerase subunits Est1 and Est3 (Fig. 2), which are required in vivo for full telomerase function [5]. Consequently, no telomere addition can be monitored (Fig. 5B). But the binding of Est2 to NTBS increased upon IR treatment and under these conditions even Est1 and Est3 bind to NTBS (Fig. 5C–E). Interestingly, one single break induced by a HO endonuclease is not sufficient to enhance Est2-binding and no telomere addition was detectable (Fig. 5B, F). However, after IR treatment NTBS show telomere addition at 10–15% whereas no telomere addition is monitored at the N80 control region (Fig. 5B). Our data agree with studies in which multiple novel telomere addition sites were identified after DNA damage [24, 25]. In the first study, internal regions in the yeast genome were identified as the site of repair-associated telomere addition (SiRTA). In the second study, deep sequencing of yeast cells with an overload of DNA damage revealed novel sites of telomere addition. In general, uncontrolled telomere addition is regulated by the Pif1 helicase in yeast [28, 29, 31, 86]. Without Pif1 multiple telomere additions sites can be detected within internal regions [28]. NTBS sites overlap significantly with Pif1-binding sites (Additional file 2: Fig. S1D), but Est2-binding is not restricted by the presence of Pif1 (Additional file 6: Fig. S4A). We speculated that only 10–15% telomere addition were measured at NTBS in the telomere addition assay, because cells still have a functional Pif1 helicase, which prevents telomerase action to a certain extend.

**Fig. 6:**
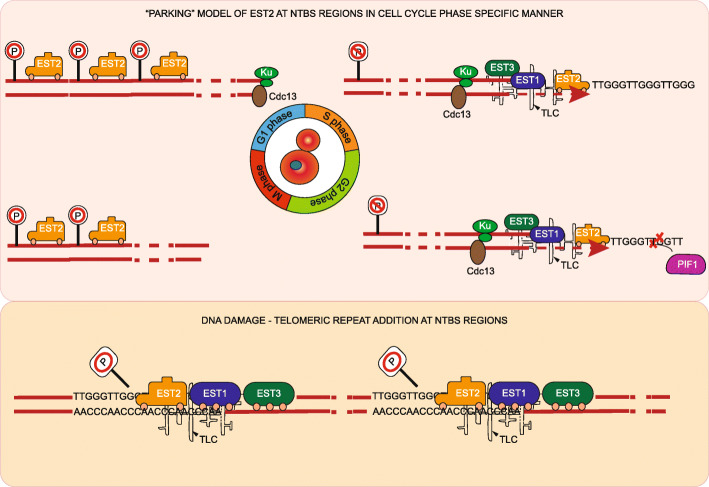
Parking model of Est2 at NTBS regions in cell cycle and DNA damaging condition. Est2 is parked at multiple internal regions, termed as NTBS (denoted by “parking” sign) within the genome in G1 and G2 phases. In S-phase, Est2 forms an active telomerase unit with Est1, Est3, and Tlc1 template along with recruitment factors Cdc13 and Ku70. Pif1, a helicase, can negatively regulate the telomerase activity. Under conditions of DNA damage, this parking is disrupted and telomerase subunit Est2, misrecognizes the breaks at NTBS regions as ends of chromosomes, and adds telomeric repeats to these regions, causing genome instability

Our study provides a comprehensive global occupancy map of yeast telomerase and presents a panel of sites at which telomere addition is prone to occur upon DNA damage (Fig. 6). Our data suggest a model in which under normal conditions Est2 binds to telomeres in S phase and to NTBS during G1 and G2 phase. Est2 binding to NTBS is supported by the 3D organization of the chromatin. Under unchallenging conditions, Est2 is inactive (parked) and no telomere addition occurs at internal sites (Fig. 6). Upon global DNA damage, Est1 and Est3 joint Est2 at NTBS and telomere addition occurs, and genome instability is enhanced.

## Conclusions

Telomere addition within the genome is observed in human cancer and congenital disorders [18, 89–91]. Telomerase-associated signatures in cancer and subtypes reveal that telomerase is not limited to ends of chromosomes but has additional functions [62–65, 67, 92–94]. Our work provides a genomic map of potentially vulnerable internal sites where telomerase subunits can bind. We reveal a novel mechanism of how telomerase is regulated in a 3D context and distinguishes between internal telomeric regions and chromosome ends. Further, the here-presented data give new insights related to genome stability and indicates certain internal regions that are more prone for telomere addition than other sites. The observation that the three-dimensional organization of telomeres alters during the cell cycle and that this organization is distorted in cancer cells [95–97], leads to the speculation that a similar mechanism also exists in higher eukaryotes.

## Methods

### Strains, plasmids, and media

All yeast strains, primers, and plasmids used in the study are listed in Additional files 8, 9, 10: Table S3, S4, and S5, respectively. Proteins were epitope-tagged at their internal loci using TRP as a marker with thirteen Myc epitopes unless stated otherwise [98]. All the strains were grown in standard YPD media under standard conditions. The epitope tagging and deletions were confirmed using PCR and sequencing before performing subsequent experiments. The strains with Est2-G8-myc in *tlc1Δ*, *est1Δ*, *est3Δ* were a generous gift from the Zakian lab. All these diploid strains were sporulated and freshly dissected spores of desired genotypes were used for ChIP analyses. RNH1 plasmid for overexpression of RNase H1 was a kind gift from Brian Luke lab.

### Chromatin immunoprecipitation (ChIP) and qPCR

ChIP experiments were performed as described previously [7]. Briefly, yeast strains were grown to OD of 0.4–0.6 and crosslinked with 1% (v/v) formaldehyde for 5 min followed by quenching of the crosslinker with the addition of 125 mM glycine. Cells were centrifuged and washed once with HBS buffer and with ChIP lysis buffer. The pellet was resuspended in ChIP lysis buffer and snap-chilled in liquid nitrogen and stored in −80°C. Frozen cell pellets were thawed, and cells were lysed using glass beads in a FastPrep (MP Biomedicals) in two rounds (60 s followed by 30 s with incubation on ice for 4 min). Chromatin was sheared to 200–1000 bp using Bioruptor Pico (Diagenode) with these settings: high intensity, 30 s ON, 30 s OFF, 7 cycles. Shearing quality was estimated on an agarose gel. Eight-microgram c-Myc antibody (Clontech) was added to the sheared chromatin and incubated at 4°C for 1 h followed by incubation with 80 μl Dynabeads protein G (Invitrogen) for 2 h. Beads were washed sequentially with SDS buffer, high-salt buffer, Tris-Lithium buffer, and Tris-EDTA buffer to remove non-specific bound DNA. Immunoprecipitated DNA was eluted using Tris-EDTA +1% SDS followed by incubation at 65°C to reverse the crosslink. Immunoprecipitated DNA was purified using Qiagen PCR purification kit and used for subsequent analyses. qPCR was performed using SyBr Green (Roche) and fold enrichment of binding regions was quantified using IP/Input method normalized to ARO1 (non-specific binder) values. Prism7 (GraphPad) was used to plot the graphs and the *p*-value was calculated using Student’s *t*-test. For IR treatments, the cells were subjected to 20 Gy of IR and allowed to recover for 30 min at 30°C before being subjected to crosslinking and ChIP.

### Telomere healing assay

Telomere addition events were quantified as described previously [26, 30]. Yeast cultures were grown overnight in XY media (10 g l^−1^ yeast extract, 20 g l^−1^ bactopeptone, 0.1 g l^−1^ adenine, 0.2 g l^−1^ tryptophan) + 2% glucose to log phase and subcultured into XY + raffinose (2%) for overnight growth. Fifteen micrograms per milliliter nocodazole was added to the cells to a density of 5–7.5 × 10^6^ cells ml^−1^ for 2 h to synchronize cells in the G2/M phase. 3% galactose was added to induce HO endonuclease expression in the strains and samples were collected after 4 h of galactose induction. Cells were plated on XY+glucose plates before and after induction of HO endonuclease and grown for 2–3 days. The total number of colonies were counted, and colonies were replica-plated to media without lysine and media with α-aminoadipic acid (α-AA) to identify the cells which have lost the distal *LYS2* gene on chromosome VII. The frequency of telomere addition was calculated as the percentage of colonies that were α-AA-resistant after HO induction. For IR experiments, the cells were exposed to 20 Gy after galactose induction and recovered for 2 h.

### Cell cycle analysis and chromatin immunoprecipitation (ChIP)

Cell synchrony experiments were performed as described previously [41]. Briefly, 320-ml yeast culture at an OD of 0.15 was arrested in G1 phase using alpha factor at a concentration of 5 μg ml^−1^ for 3–4 h. The cells were examined microscopically for shmoos. Cells were filtered and resuspended in fresh YPD media and released into YPD+pronase at 24°C. Samples were collected after every 15 min for FACS analysis and cross-linking was performed using the conditions mentioned above. FACS samples were spun down and fixed in 70% (v/v) ethanol overnight at 4°C. Cells were washed with 50 mM Tris buffer (pH 7.8) followed by RNase digestion for 5 h at 37°C and proteinase K digestion for 60 min at 50°C. Cells were sonicated with low intensity (30 s on, 30 s off, 3 cycles) to break clumps and incubated with SYTOX Green before being subjected to FACS analysis. FACS data was analyzed with FlowJo (BD).

### ChIP-chip

ChIP was performed as described above and for genome-wide analysis, immunoprecipitated DNA was amplified, labeled with minor modifications of Agilent Yeast ChIP on chip protocol v9.2. Binding sites were identified using ChIPOTle 2.0 [99] and corrections were applied to control for the false discovery rate as described in [41]. The identified sites and their location within the genome are listed in Additional file 1: Table S1.

### Hi-C methods

The Hi-C protocol used here was amended from the Hi-C 2.0 [100] to yeast cells. Briefly, *S. cerevisiae* diploid cells were grown in YPD (1% yeast extract, 2% peptone, 2% glucose) to exponential phase, and 100 ml of cells (50–80 OD, sufficient for 1 Hi-C library) was fixed with formaldehyde at 3% final concentration for 20 min at 30°C, 250 rpm, and quenched by incubating with a final concentration of 0.35 M glycine (2× the volume of formaldehyde added) for an additional 5 min. Cells were washed with water and pellets were snap frozen and stored at −80°C. Cells were thawed, washed in spheroplasting buffer (SB, 1 M sorbitol, 50 mM Tris pH 7.5), and digested with 10 μg ml^−1^ Zymolyase 100T in SB containing 0.5% beta-mercaptoethanol for 10 min at 35°C. Cells were washed in restriction enzyme buffer (NEB3.1) and chromatin was solubilized by adding SDS to 0.01% and incubating at 65°C for 5 min. Excess SDS was quenched by addition of Triton X100 to 1%. Chromatin was incubated with 400 U of DpnII overnight at 37°C and 400 rpm. DpnII was inactivated by incubation at 65°C for 20 min and DNA ends were filled-in with nucleotides substituting dCTP for biotin-14-dCTP using Klenow fragment DNA polymerase I at 23°C for 4 h in a thermomixer (900 rpm mixing for 10 s every 5 min). The sample volume was diluted 2-fold and crosslinked DNA ends ligated at 16°C for 4 h using 50 U of T4 DNA ligase in 1x T4 ligation buffer (Invitrogen), 1% Triton and 0.1 mg ml^-1^ BSA.

Crosslinks were reversed overnight at 65°C in the presence of proteinase K (400 μg ml^−1^) and an additional 2 h with another addition of proteinase K (400 μg ml^−1^). DNA was purified by phenol:chloroform:isoamylalcohol (25:24:1) extraction and precipitated with 2.5 vol ethanol, dissolved in TE (10 mM Tris pH 8.0, 0.1 mM EDTA) and washed and concentrated with an Amicon 30 kDa column, before treating with 10 μg/ml of RNase A at 37°C for 30 min. Biotin was removed from unligated ends by incubation with 0.3 U μl^−1^ T4 DNA polymerase and low abundance of dATP and dGTP (25 μM each) at 20°C for 4 h and at 75°C for 20 min for inactivation of the enzyme. DNA was washed on an Amicon 30 kDa column and subsequently fragmented using a Covaris M220 ultrasonicator (duty factor 20%, 200 cycles/burst, 240 s, 20°C). DNA ends were repaired using T4 DNA polymerase, T4 polynucleotide kinase, and Klenow fragment DNA polymerase I. Biotinylated fragments were enriched using Streptavidin C1 magnetic beads (Invitrogen). DNA ends were A-tailed and NextFlex (Bioo Scientific) barcoded adapters were ligated while the DNA was on the beads. Resulting libraries were minimally amplified by PCR and sequenced using paired end 75 bp reads on a NextSeq550 (Illumina).

### Hi-C bioinformatic analyses

We performed iteration analyses to quantify the overlap between NTBS sites and genomic features such as ORFs, Pif1-binding sites and DNA damage sites. We then mapped the sequencing reads to the yeast genome using the HiCUP pipeline [101]. Statistical analysis of the telomere proximal ends was performed using custom R scripts and significance of the results was determined by non-parametric Wilcoxon-rank tests.

### Generation of Hi-C contact maps

Paired-end sequencing reads were mapped independently to the genome of *S. cerevisiae* S288C (NCBI Primary Assembly: GCF_000146055.2) using Bowtie 2.3.5 [102] and an algorithm which iteratively increases truncation length to maximize yield of valid Hi-C interactions. Only read pairs with both reads uniquely aligned to the genome were considered for subsequent steps. The S*. cerevisiae* genome was then divided into restriction fragments produced by the restriction enzyme DpnII. Each read of a read pair was sorted into its corresponding restriction fragment. Read pairs were classified as valid Hi-C products, non-ligation or self-ligation products; only valid Hi-C products were considered below.

To create interaction matrices, the *S. cerevisiae* genome was first divided into bins of length 10 kbp. We then assigned valid Hi-C products to the bins proportional to their overlap, i.e., each read contributes a count of one to the contact map, but it can be split between bins. As raw Hi-C contact frequency maps are biased due to the uneven distribution of restriction enzyme sites, differences in GC content, and the mappability of individual reads, we normalized raw contact maps using the Sinkhorn-Knopp balancing algorithm. Resulting matrices were normalized so that Hi-C scores for each row and column sum to 1. Subsequent analysis and visualization were done using Python and R scripts. (http://projecteuclid.org/euclid.pjm/1102992505).

To quantify NTBS-NTBS vs. NTBS-telomeres interactions, we assigned NTBS sites and telomeres to the respective bin of the Hi-C contact matrix of wild-type *S. cerevisiae* and collected the respective Hi-C contact probabilities. We then checked for each NTBS site whether its contact probability with one of the two telomeres is higher than the mean contact probability with all the other NTBS sites. This analysis yielded that NTBS sites are closer to each other (86%) than to telomeres (14%).

## Supplementary Information

**Additional file 1: Supplementary Table S1.** Genomic coordinates of NTBS regions discovered in at least three out of five independent ChIP-chip experiments.

**Additional file 2: Supplementary Figure S1. A** Snapshots of IGV browser showing the presence of NTBS #1-4 in the yeast genome. **B-F** Bioinformatics’ analyses demonstrating the overlap of genomic features with the NTBS regions. P-value denotes statistical significance of their enrichment in the NTBS set between the features and NTBS regions. In **B** NTBS vs. G4s, **C** NTBS vs R-loops, **D** NTBS vs. Pif1-binding sites, **E** NTBS vs. DNA Pol2 sites *in pif1-m2* cells, **F** NTBS vs. DNA Pol2 sites and **G** NTBS vs. γ-H2A-binding sites significantly overlapped with NTBS regions.

**Additional file 3: Supplementary Table S2.** Table defining the NTBS overlap with gene bodies, percentage overlap of NTBS region and the gene and gene function description.

**Additional file 4: Supplementary Figure S2.** Est2-binding to NTBS regions in absence of telomerase components TLC1 and Est1. **A** Cell cycle progression was monitored with flow cytometry and FACS analysis demonstrated the cell cycle stage of synchronized cells in wild type, *tlc1Δ* and *est1Δ*. **B-D** Est2-binding to NTBS #2-#4 in wild type (closed circles), *tlc1Δ* (open squares) and *est1Δ* (open triangles). A reproducible increase of Est2- NTBS-binding was observed in absence of tlc1 and est1 in independent replicates. The data plotted are standard mean ± SEM for n = 3 replicates.

**Additional file 5: Supplementary Figure S3.** Canonical telomerase recruitment factors, Cdc13 and Ku7 0, do not bind to NTBS. **A** Cdc13-binding to four NTBS (#1-#4) and telomere VI-R was monitored by ChIP-qPCR in synchronous cultures. ChIP analysis of Cdc13 in different cell cycle stages did not show enrichment to NTBS regions. Data plotted are mean ± SEM) normalized to ARO1 levels at respective timepoints. **B** Cell cycle analysis was determined using flow cytometry. Representative graphs demonstrating different cell cycle stages after release from α-factor. **C** Cdc13 and **D** Ku70-binding to NTBS regions in undamaged (light grey bars) and damaging conditions (IR, dark grey bars). No statistically significant enrichment of Cdc13 and Ku70 was observed to NTBS regions. Data represented are mean ± standard error for n = 3 biological replicates. Statistical significance compared to untreated cells were determined using Student’s t-test. * p-value < 0.05.

**Additional file 6: Supplementary Figure S4.** Est-NTBS interaction is independent of regulatory factors, Pif1, Mlh1, R-loops, heterochromatin stage and Sir4. Est2-binding to NTBS regions (NTBS#1-#4) in wild type (grey bars) and absence of regulatory factors (white bars) **A** Est2-NTBS-binding was evaluated using ChIP-qPCR in *pif1-m2* cells that express reduced nuclear Pif1, negative regulator of telomerase. No significant change was observed in *pif1-m2* cells (dark grey bars) compared to wild type condition (light grey bars). **B** R-Loops were resolved using overexpression of RNAseH1 plasmid (RNH1) (dark grey bars) compared to wild type condition (light grey bars). **C** ChIP-qPCR of Est2-NTBS-binding in *mlh1∆* (suppressor of genomic telomere insertions) cells (dark grey bars) compared to wild type condition (light grey bars). **D** Est2-NTBS interaction in *sir4∆* cells (dark grey bars) compared to wild type condition (light grey bars). No statistically significant enrichment to NTBS sites were observed for all the tested conditions. **E** Est2-NTBS interaction in *sin3∆* (component of histone deacetylase complexes) cells (dark grey bars) compared to wild type condition (light grey bars). No statistically significant enrichment to NTBS sites were observed for all the tested conditions. Data represented are mean ± SEM. Statistical significance was calculated in comparison to ARO1 levels for n = 3 biological replicates and determined using Student's t-test. No statistically significant enrichment to NTBS sites were observed for all the tested conditions.

**Additional file 7: Supplementary Figure S5.** HR connection to Est2-NTBS interaction and schematic of telomere healing assay. **A** Est2-binding to NTBS regions in wild type (light grey bars) and in absence of Rad52 (dark grey bars). Bars represent mean ± standard error mean for n = 3 biological replicates. The significance was calculated between wild type and *rad52Δ* cells using Student’s t-test. * p-value < 0.05. **B** Telomere healing assay description. NTBS regions cloned adjacent to HO site were subjected to HO cleavage to create a double stranded break. Lysine (LYS2) marker was lost upon telomere addition and retained if the break was repaired.

**Additional file 8: Supplementary Table 3.** List of bacterial strains used in this study.

**Additional file 9: Supplementary Table 4.** List of yeast strains used in this study.

**Additional file 10: Supplementary Table 5.** List of primers used in this study.

## Data Availability

The datasets generated during this study are available at Gene Expression Omnibus (GEO) GSE143187 [103].

## Abbreviations

ARS: Autonomously replicating sequence
ChIP: Chromatin immunoprecipitation
DNA: Deoxyribonucleic acid
DSB: Double-strand break
FACS: Fluorescence-activated cell sorting
G4: G-quadruplex
GCR: Gross chromosomal rearrangement
HR: Homologous recombination
IR: Ionizing gamma radiation
MRX: Mre11-Rad50-Xrs2
NTBS: Non-telomeric binding sites
ORF: Open reading frames
RNA: Ribonucleic acid
SGD: *S. cerevisiae* genome database
SiRTAs: Sites of repair-associated telomere addition
TERRA: Telomeric repeat-containing RNA
TPE: Telomere position effect
