# Author Correction to: Telomerase subunit Est2 marks internal sites that are prone to accumulate DNA damage

**DOI:** 10.1186/s12915-022-01237-y

**Published:** 2022-02-01

**Authors:** Satyaprakash Pandey, Mona Hajikazemi, Theresa Zacheja, Stephanie Schalbetter, Matthew J. Neale, Jonathan Baxter, Victor Guryev, Andreas Hofmann, Dieter W. Heermann, Stefan A. Juranek, Katrin Paeschke

**Affiliations:** 1 University of Groningen, University Medical Center Groningen, European Research Institute for the Biology of Ageing, 9713 AV Groningen, Netherlands; 2 Clinic of Internal Medicine III, Oncology, Hematology, Rheumatology and Clinical Immunology, University Hospital Bonn, Bonn, Germany; 3 Department of Life Science, University of Sussex, Brighton, UK; 4 Institute for Theoretical Physics, University of Heidelberg, Philosophenweg 12, 69120 Heidelberg, Germany; 5 Clinic of Internal Medicine III, Oncology, Hematology, Rheumatology and Clinical Immunology, University Hospital Bonn, Bonn, Germany; 6 University of Groningen, University Medical Center Groningen, European Research Institute for the Biology of Ageing, 9713 AV Groningen, Netherlands

**Correction to: BMC Biol 19, 247 (2021)**

**https://doi.org/10.1186/s12915-021-01167-1**

The original article [1] mistakenly omitted co-author Matthew J Neale and his funding source.

This has since been corrected.
