# Supplementary figures and images for: Telomerase subunit Est2 marks internal sites that are prone to accumulate DNA damage
Source: BMC Biol. 2021 Nov 20;19:247. doi: 10.1186/s12915-021-01167-1 (PMC8605574; doi:10.1186/s12915-021-01167-1)

A

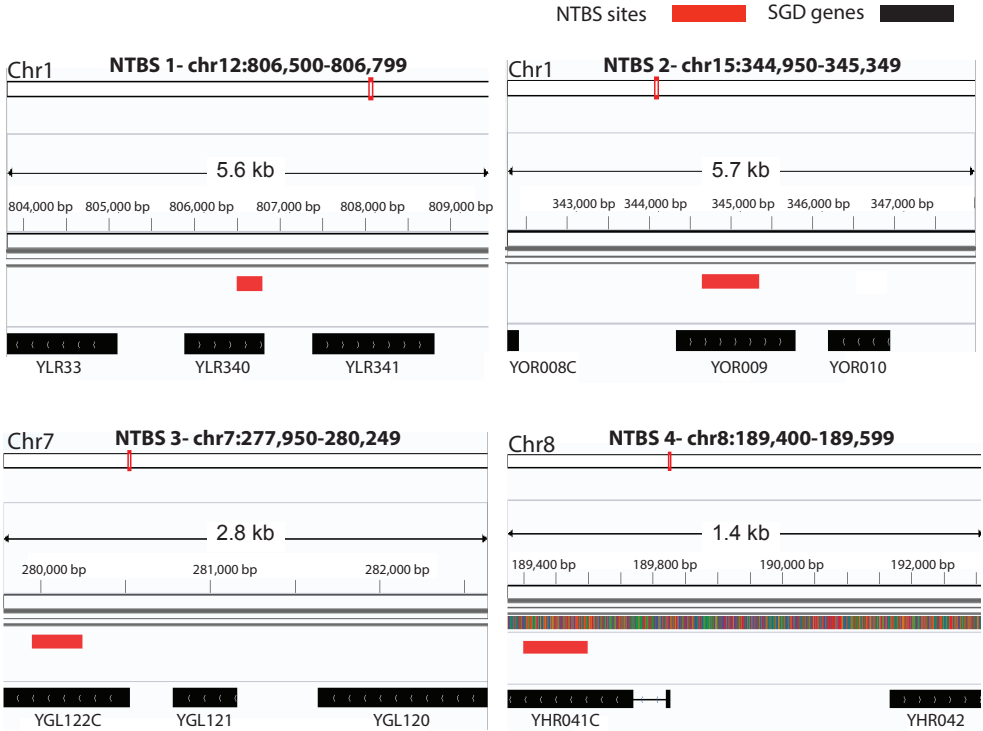

B

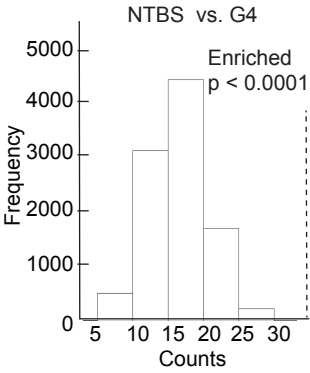

C

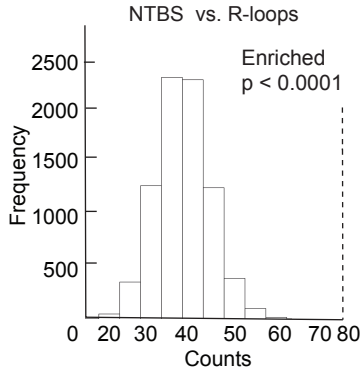

D

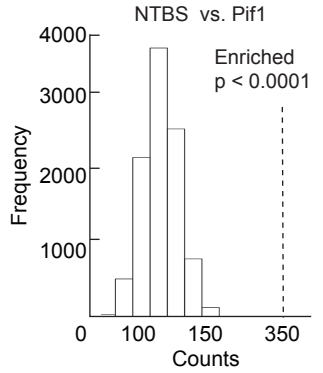

E

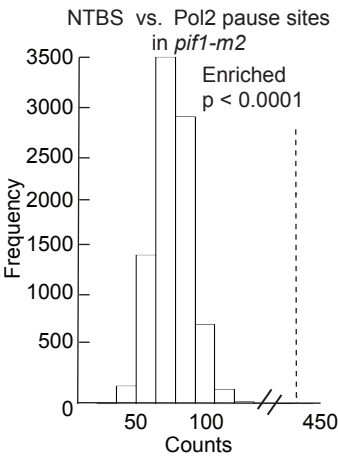

F

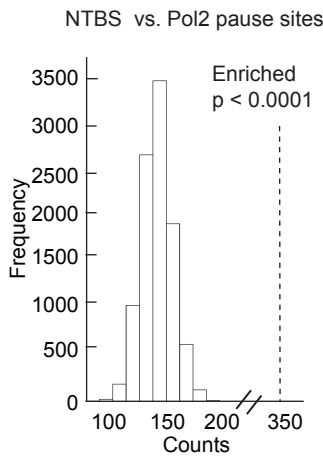

G

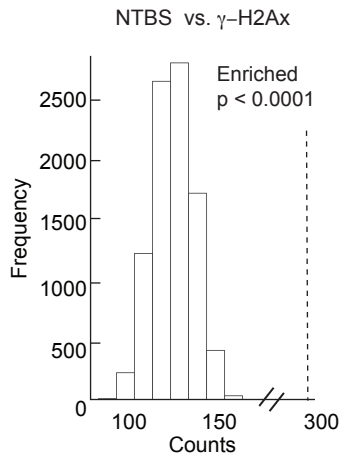

Supplement: Supplementary file 2 — Additional file 2: Supplementary Figure S1. A Snapshots of IGV browser showing the presence of NTBS #1-4 in the yeast genome. B-F Bioinformatics’ analyses demonstrating the overlap of genomic features with the NTBS regions. P-value denotes statistical significance of their enrichment in the NTBS set between the features and NTBS regions. In B NTBS vs. G4s, C NTBS vs R-loops, D NTBS vs. Pif1-binding sites, E NTBS vs. DNA Pol2 sites in pif1-m2 cells, F NTBS vs. DNA Pol2 sites and G NTBS vs. γ-H2A-binding sites significantly overlapped with NTBS regions. [file 12915_2021_1167_MOESM2_ESM.pdf]

**A**Est2-myc wild type Est2-myc in *tlc1* $\Delta$  Est2-myc in *est1* $\Delta$ 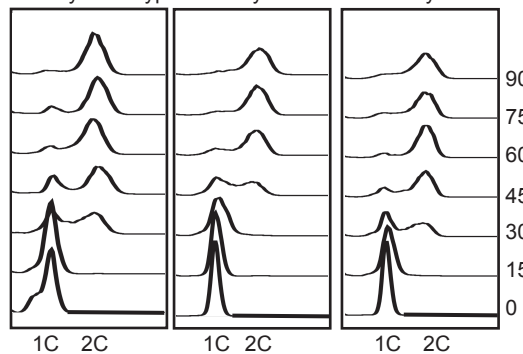**B**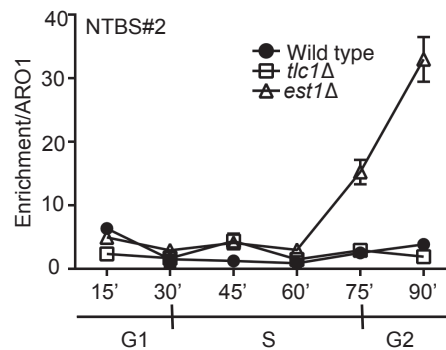**C**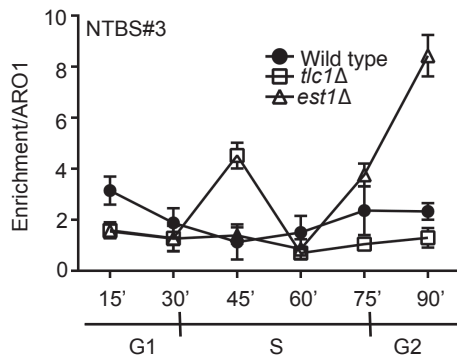**D**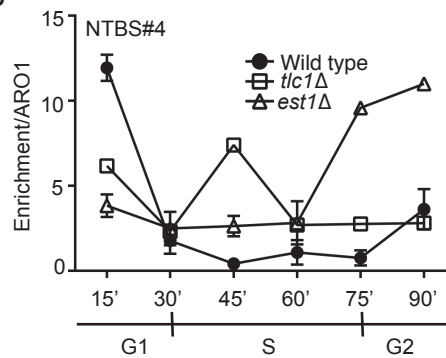

Supplement: Supplementary file 4 — Additional file 4: Supplementary Figure S2. Est2-binding to NTBS regions in absence of telomerase components TLC1 and Est1. A Cell cycle progression was monitored with flow cytometry and FACS analysis demonstrated the cell cycle stage of synchronized cells in wild type, tlc1Δ and est1Δ. B-D Est2-binding to NTBS #2-#4 in wild type (closed circles), tlc1Δ (open squares) and est1Δ (open triangles). A reproducible increase of Est2- NTBS-binding was observed in absence of tlc1 and est1 in independent replicates. The data plotted are standard mean ± SEM for n = 3 replicates. [file 12915_2021_1167_MOESM4_ESM.pdf]

**A**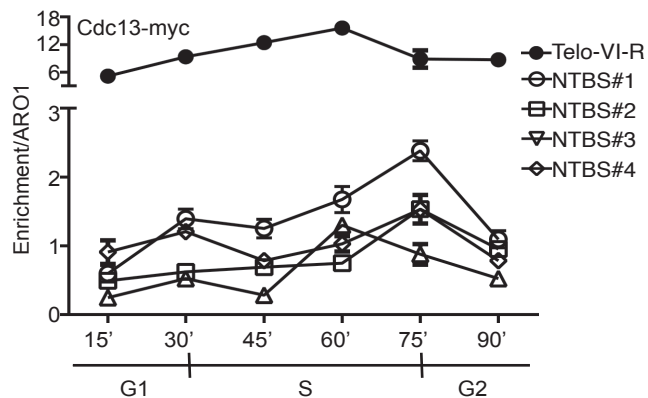**B**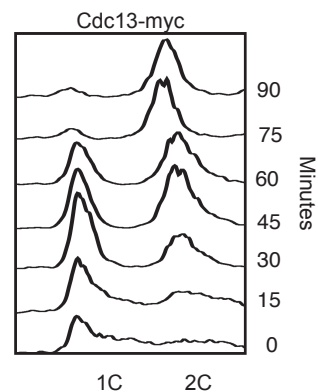**C**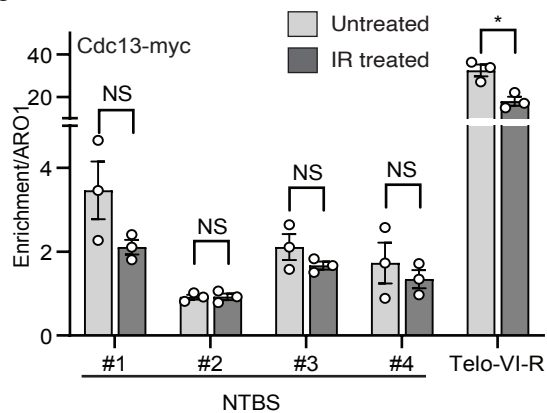**D**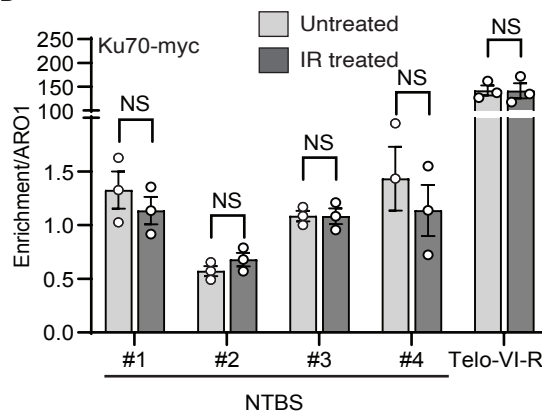

Supplement: Supplementary file 5 — Additional file 5: Supplementary Figure S3. Canonical telomerase recruitment factors, Cdc13 and Ku7 0, do not bind to NTBS. A Cdc13-binding to four NTBS (#1-#4) and telomere VI-R was monitored by ChIP-qPCR in synchronous cultures. ChIP analysis of Cdc13 in different cell cycle stages did not show enrichment to NTBS regions. Data plotted are mean ± SEM) normalized to ARO1 levels at respective timepoints. B Cell cycle analysis was determined using flow cytometry. Representative graphs demonstrating different cell cycle stages after release from α-factor. C Cdc13 and D Ku70-binding to NTBS regions in undamaged (light grey bars) and damaging conditions (IR, dark grey bars). No statistically significant enrichment of Cdc13 and Ku70 was observed to NTBS regions. Data represented are mean ± standard error for n = 3 biological replicates. Statistical significance compared to untreated cells were determined using Student’s t-test. * p-value < 0.05. [file 12915_2021_1167_MOESM5_ESM.pdf]

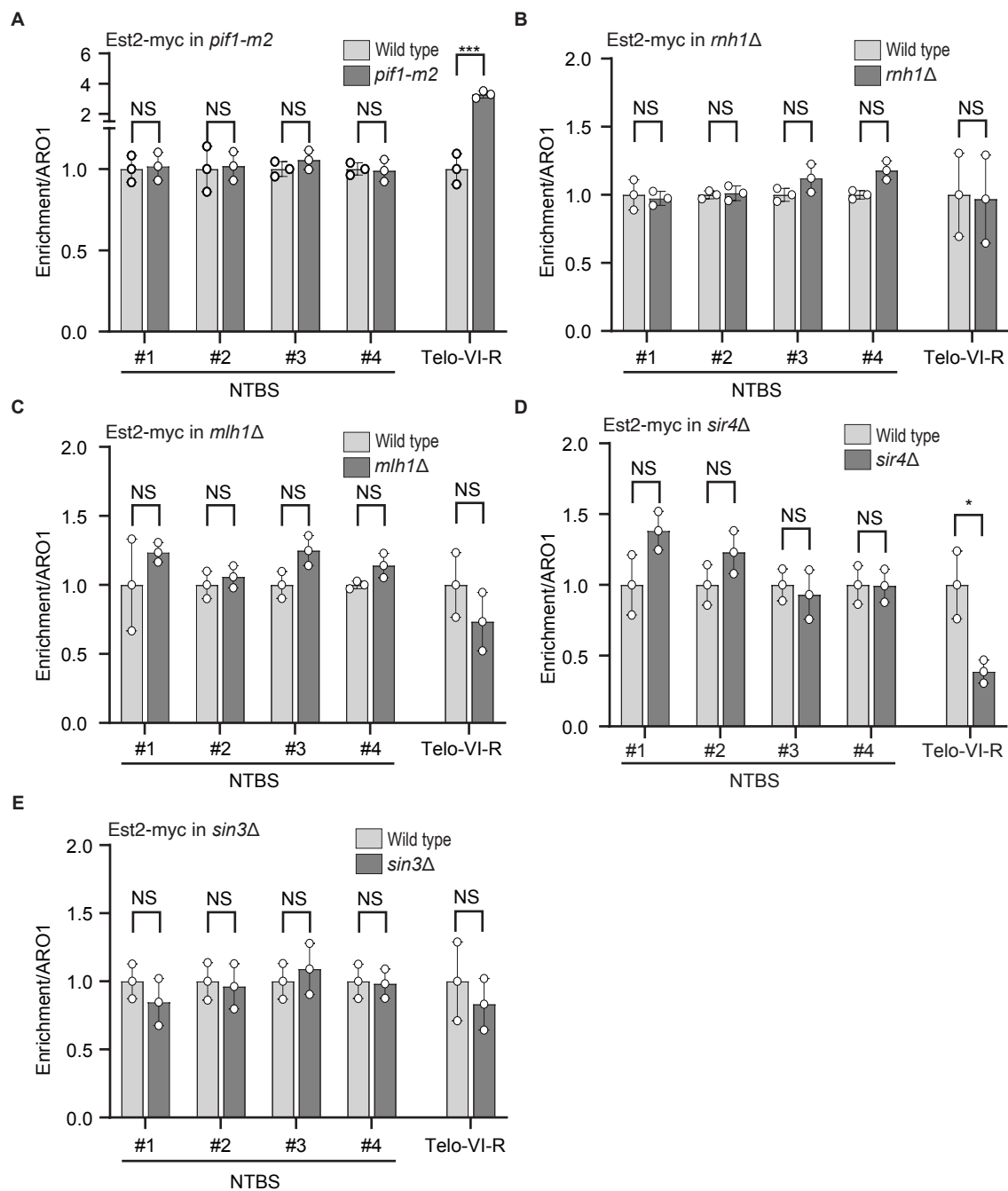

Supplement: Supplementary file 6 — Additional file 6: Supplementary Figure S4. Est-NTBS interaction is independent of regulatory factors, Pif1, Mlh1, R-loops, heterochromatin stage and Sir4. Est2-binding to NTBS regions (NTBS#1-#4) in wild type (grey bars) and absence of regulatory factors (white bars) A Est2-NTBS-binding was evaluated using ChIP-qPCR in pif1-m2 cells that express reduced nuclear Pif1, negative regulator of telomerase. No significant change was observed in pif1-m2 cells (dark grey bars) compared to wild type condition (light grey bars). B R-Loops were resolved using overexpression of RNAseH1 plasmid (RNH1) (dark grey bars) compared to wild type condition (light grey bars). C ChIP-qPCR of Est2-NTBS-binding in mlh1∆ (suppressor of genomic telomere insertions) cells (dark grey bars) compared to wild type condition (light grey bars). D Est2-NTBS interaction in sir4∆ cells (dark grey bars) compared to wild type condition (light grey bars). No statistically significant enrichment to NTBS sites were observed for all the tested conditions. E Est2-NTBS interaction in sin3∆ (component of histone deacetylase complexes) cells (dark grey bars) compared to wild type condition (light grey bars). No statistically significant enrichment to NTBS sites were observed for all the tested conditions. Data represented are mean ± SEM. Statistical significance was calculated in comparison to ARO1 levels for n = 3 biological replicates and determined using Student's t-test. No statistically significant enrichment to NTBS sites were observed for all the tested conditions. [file 12915_2021_1167_MOESM6_ESM.pdf]

**A**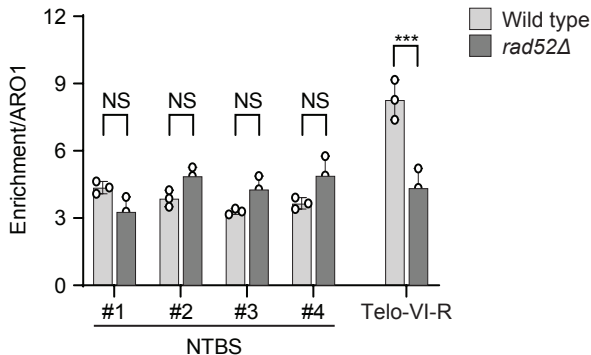**B**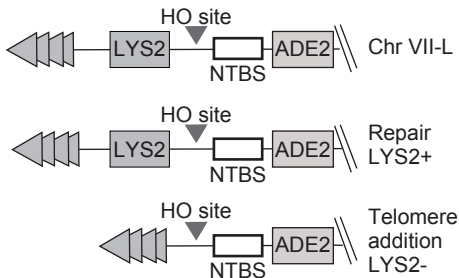

Supplement: Supplementary file 7 — Additional file 7: Supplementary Figure S5. HR connection to Est2-NTBS interaction and schematic of telomere healing assay. A Est2-binding to NTBS regions in wild type (light grey bars) and in absence of Rad52 (dark grey bars). Bars represent mean ± standard error mean for n = 3 biological replicates. The significance was calculated between wild type and rad52Δ cells using Student’s t-test. * p-value < 0.05. B Telomere healing assay description. NTBS regions cloned adjacent to HO site were subjected to HO cleavage to create a double stranded break. Lysine (LYS2) marker was lost upon telomere addition and retained if the break was repaired. [file 12915_2021_1167_MOESM7_ESM.pdf]
